# O005: Results of the french national audit on standard precautions

**DOI:** 10.1186/2047-2994-2-S1-O5

**Published:** 2013-06-20

**Authors:** M Giard, E Laprugne-Garcia, E Caillat-Vallet, I Russell, D Verjat-Trannoy, M-A Ertzscheid, N Vernier, C Laland, A Savey

**Affiliations:** 1 CClin Sud-Est, Lyon, France; 2 UMR5558, LBBE, Lyon 1 University, Villeurbanne, France; 3 CClin Paris-Nord, Paris, France; 4 CClin Ouest, Rennes, France; 5 CClin Est, Nancy, France; 6 CClin Sud-Ouest, Poitiers, France

## Introduction

Standard precautions (SP) aim to protect healthcare workers (HCW) and patients from infectious diseases arising from bloodborne pathogens and reduce the risk of cross transmission of micro-organisms. They must be applied in all circumstances, regardless of the infectious status of the patient.

## Objectives

The objectives were to assess: 1) institutional policies for SP promotion; 2) available resources for SP implementation; and 3) education of HCW and their compliance with SP.

## Methods

The study was a mixed audit of procedures, resources and attitudes. It was conducted between February 1^st^ and December 31^st^ 2011, supported by the Ministry of Health. Inclusion criteria were voluntary public and private hospitals in France, medical, surgical and medico-technical wards therein and HCW working with patients in these wards. Self-assessment questionnaires were administered at three levels: institutional, ward and HCW. At institutional and ward levels, results were given as a percentage of objectives attained; at professional level, percentages of responses reported as “never”, “sometimes”, “often” or “always” were calculated for each question.

## Results

A total of 1,599 hospitals participated, including 14,968 wards and 203,840 HCW. At institutional level, the overall score was 88%, covering: SP promotion (91%), procedures (99%) and SP evaluation (63%). At ward level, the overall score was 94%, covering: procedures (95%) and resources (93%). Among the 165,722 (81.3%) HCW who reported having participated in a training session on SP, 69.6% had had it in the last five years. A total of 88.1% of HCW knew where to find the appropriate written procedure in the event of a blood exposure. HCW reported the best compliance for glove changing between two patients (94.5% “always”). The less respected criteria were glove use for intramuscular or subcutaneous injection and eye protection use in the event of blood exposure risk (34.5% and 24.4% “always”, respectively).

## Conclusion

No study on SP exists in literature which includes such a large participation as this one. It will form a refence basis leading to actions for improvement at local and national level.

## Disclosure of interest

None declared.

